# Editorial: Clinical aspects of obstructive sleep apnea and cardiovascular consequences

**DOI:** 10.3389/fneur.2022.961240

**Published:** 2022-10-06

**Authors:** Kittisak Sawanyawisuth, Shazia M. Jamil, Melissa C. Lipford

**Affiliations:** ^1^Department of Medicine, Faculty of Medicine, Khon Kaen University, Khon Kaen, Thailand; ^2^Division of Pulmonary, Critical Care and Sleep Medicine, Department of Medicine, Scripps Clinic, La Jolla, CA, United States; ^3^Department of Neurology, Center for Sleep Medicine, Mayo Clinic, Rochester, MN, United States

**Keywords:** sleep apnea, continuous positive air pressure (CPAP), cardiovascular diseases, compliance, risk factor

This article is comprised of two parts. The first part is a review of obstructive sleep apnea (OSA) including epidemiological data, risk factors, diagnosis, and treatment on cardiovascular consequences. The second part is a summary of articles published in the Research Topic: Clinical Aspects of Obstructive Sleep Apnea and Cardiovascular Consequences. Note that this review is mainly focused on OSA. Some data may include the obstructive sleep apnea syndrome (OSAS) which defined by OSA with respiratory disturbance index (RDI) > 14 events/hour, or OSA with RDI > 5 associated with symptoms or cardiovascular diseases (1).

## OSA and cardiovascular consequences: A clinical review

OSA is a common disease in clinical practice and is often undiagnosed. In the general population, the prevalence of OSA can be as high as 38% (2), however sharply inclines to up to 78 to 90% in elderly women and men, respectively (2). A population study in Australia noted a high prevalence in middle-age men and women (57.7 and 41.7%, respectively) (3). For those admitted with cardiovascular disease or patients with cerebrovascular disease, the prevalence of OSA in both conditions was also high at 48% and 70.4% (4, 5).

There are several risk factors of OSA which can be classified into four categories: body mass index, age and sex, anatomical risks, and co-morbid diseases. First, obesity is a well-known risk factor for OSA. Approximately 45% of obese patients were found to have OSA (6), and the prevalence was higher in those undergoing bariatric surgery at 77.2% (7). In contrast, 60% of patients with OSA were obese in Western countries (8), while only 36.6% of patients with OSA in the Eastern countries were obese (9). Note that criterion to define obesity is different (30 vs. 25 kg/m^2^) in different populations. Secondly, older age, male sex and post-menopausal women are all risk factors for OSA. The prevalence of OSA increases from 10% to 17% in male patients as age group increases from 30–49 years to 50–70 years with an apnea-hypopnea index (AHI) of 15 events/hour or more (10). In adult patients, male:female ratio appears to be 4.9:1 (11), however narrows to 1:1 in post-menopausal women (12). Thirdly, craniofacial abnormalities that lead to narrow oropharyngeal space such as torus palatinus, torus mandibularis, macroglossia, tonsillar enlargement, microretrognathia, or edentate status are risk factors (13, 14). Finally, certain co-morbid diseases may be related to OSA such as hypothyroidism, chronic kidney disease, HIV infection, epilepsy, asthma, chronic obstructive airway disease, acromegaly, and allergic rhinitis (15–20).

Diagnosis of OSA can be made by several devices. The gold standard is polysomnography type 1 with evidence of AHI or RDI of five or more events/hour. Note that AHI and RDI are different: AHI defined by an average number of apnea and hypopnea events per hour of sleep, while RDI is number of apnea and hypopnea events plus number of respiratory effort-related arousals per hour of sleep (21). Other types of polysomnography such as type 2 and 3 are also acceptable as they have sensitivity and specificity of 80% or over. A study from China found that home sleep test had comparable respiratory event index (REI), an AHI for home sleep test (22), with an AHI by in-lab polysomnography (*p* = 0.103). Both REI by home sleep test and AHI by in-lab polysomnography had a good correlation with a correlation index of 0.779; *p* < 0.001 with sensitivity, specificity, and accuracy of 94.9, 62.5, and 91.0%, respectively (23). Other home-based testing devices such as smart watch or arterial tonometry are also reported to be reliable in diagnosing OSA with sensitivity of 90.64 and 95.8%, respectively (24, 25). Note that out of center devices may only be reliable in those with moderate to severe OSA.

The mainstay treatment of OSA is a continuous positive airway pressure device (CPAP). There is significant evidence suggesting that CPAP is effective in reducing cardiovascular risk. For hypertension, CPAP therapy reduced new onset hypertension by 29% (Hazard ratio of 0.71; 95% confidence interval 0.53, 0.94) (26). Systolic and diastolic blood pressure reduction was 2.6 and 2.0 mmHg, respectively with CPAP therapy (27). Blood pressure reduction with CPAP therapy was more pronounced in patients with resistant hypertension with OSA since systolic and diastolic blood pressure reduction was 7.21 and 4.99 mmHg respectively (28). CPAP therapy in patients with heart failure has shown reduced risk of heart failure in patients 60 years and older by 17–19% and increased left ventricular ejection fraction by 5.18 (95% confidence interval 3.27, 7.08) (29, 30). In patients with concurrent OSA and atrial fibrillation, those using CPAP therapy had reduced risk of atrial fibrillation recurrence by 40% than nonusers (31, 32). Additionally, CPAP therapy could be held responsible in converting atrial fibrillation to sinus rhythm in two cases (33, 34). Patients with severe OSA were found to have lower incidence of fatal cardiovascular events when treated with CPAP than those without treatment (0.35 vs. 1.06 per 100 person-year; *p* = 0.0008) (35).

Regarding CPAP for secondary prevention in patients with established cardiovascular diseases, a study conducted in patients with sleep apnea and cardiovascular diseases did not find significant benefits of CPAP over usual care on deaths from cardiovascular diseases. The hazard ratio with CPAP was 1.10 with 95% confidence interval of 0.91 to 1.32 (36). However, later studies showed benefits of CPAP on secondary prevention of cardiovascular diseases. A meta-analysis published in 2018 found that CPAP significantly reduced risk of major cardiovascular events in patients with coronary artery disease by 39% than control by six observational studies (37). But, the results were not consistent in an analysis of two randomized controlled trials (relative risk of 0.57; *p* = 0.06). Note that there were only two randomized controlled trials included and the p value was also significant. For patients with hypertension and OSA, blood pressure was significantly reduced in those with good CPAP compliance in both patients with hypertension and resistant hypertension (38, 39). Systolic and diastolic blood pressure were lower by 3.9 and 3.5 mmHg, respectively in patients with resistant hypertension. Further studies regarding the effects of CPAP for secondary prevention of cardiovascular diseases are required.

Note that the goal of CPAP therapy is to use it regularly and throughout the duration of sleep (i.e.7–8 h per night), limiting interpretation of results of a large randomized controlled trial showing no cardiovascular benefits in a population using CPAP for an average of 3.3 h/night (36, 40). Even though CPAP is effective in treatment of OSA and its consequences, acceptance and compliance of CPAP is still problematic. Studies show that in resource limited countries, approximately half of patients with OSA agree to purchase CPAP and about half of them use CPAP regularly (41–43). An issue with insurance coverage may affect patient's decision on CPAP acceptance or purchasing. CPAP may cause some side effects such as discomfort, aerophagia, dry mouth, rhinitis, or rarely pneumothorax (44–47). Other treatments include oral appliance, weight reduction in overweight and obese patients, positional therapy, and exercise (48–50).

Mandibular advancement device (MAD), an oral appliance for OSA, is an alternative treatment for patients with mild to moderate OSA, and for patients with severe OSA who are unable to tolerate CPAP or have contraindication for CPAP such as altered level of consciousness, high aspiration risk or inability to swallow, or facial abnormalities. MAD may have benefits on lower blood pressure in women with OSA (51). After 4 months of randomized controlled trial, women wearing MAD had significantly lower mean nighttime systolic and diastolic blood pressure by 10.8 and 6.6 mmHg than the control group (*p* value 0.001 and 0.002). MAD can be used in a combination with CPAP. Possible side effects of MAD should be taken into account: tooth pain, excessive salivation, gum soreness, gingival irritation, temporomandibular joint pain, and myofascial pain. Individualized or personalized management of OSA may be warranted.

## Summation of Research Topic

OSA is a common disease with several medical consequences if left undiagnosed or untreated, which can be categorized into two groups: cardiovascular and non-cardiovascular. This Research Topic focused on clinical aspects of cardiovascular diseases including review, epidemiological, treatment-related, or interventional studies. Cardiovascular consequences of OSA included hypertension, coronary artery disease, cardiac arrhythmia, left ventricular hypertrophy, cardiac failure, and stroke (52–60).

There are eight articles recently published in this topic with 41 authors from China, Germany, Japan, USA, Brazil, Israel, and Taiwan. These articles can be classified into three groups: review (1), epidemiological study (3), and CPAP-related (4).

First, a review published by Yasir et al. emphasized if cardiovascular outcomes in sleep-disordered breathing are under-estimated? The authors summarized how clinicians conducted research in the past and present to show cardiovascular outcomes in sleep-disordered breathing particularly in OSA. The authors proposed to have diagnostic approach by using personalized approach as well as big data research, and the need for identification of patients with OSA who are at risk for cardiovascular diseases.

The three epidemiological studies published include a population-based study by dos Santos Silva, Markers of Carotid Plaque by Lavie et al., and correlation of cardinal features of OSA and blood pressure by Xia et al.. The population-based study was conducted in older adults in Brazil. The authors found that severity of OSA was significantly related with cardiovascular consequences including hypertension and heart disease. The authors also found that obesity was not related to severity of OSA in older adults. Previous studies also showed that obesity may not be related to OSA diagnosis particularly in the elderly and those with hypertension (52, 61). Xia et al. conducted a retrospective study to show that both low oxygenation and arousal during apneic events in patients with OSA were significantly related to high diastolic blood pressure and hypertension. This article emphasized roles of intermittent hypoxemia during sleep and micro awakening from OSA to high blood pressure. Lavie et al. showed that patients with OSA may already have carotid plaque destabilization even in asymptomatic patients. There were three important markers for carotid plaque abnormalities including 3-nitrotyrosine, intracellular lipids content, and smooth muscle cell-actin. This study used an arterial tonometry device to diagnose OSA (62).

Finally, we have four studies related to CPAP therapy in patients with OSA and cardiovascular consequences. First, Zhang et al. showed that only 63 patients out of 306 patients (20.59%) diagnosed with OSA accepted CPAP as the treatment. Additionally, sleep efficiency and AHI were independently associated with acceptance of CPAP treatment with an adjusted odds ratio of 1.043 (95% confidence interval of 1.02, 1.067) and 1.022 (95% confidence interval of 1.01, 1.034), respectively. (63–65). Patients with more severe OSA who are symptomatic may tend to purchase and use CPAP more regularly. The second study investigated the effect of CPAP use on weight and glycemic control in patients with Type 2 Diabetes Mellitus (DM). The study had a median duration of CPAP use of 1.6 years. Surprisingly, patients with type 2 DM and PAP treatments were found to have an increased risk of severe long-term weight gain and an increase in HbA1c. Even though there are several potential explanations of these findings, further studies evaluating long term effects of CPAP treatment are needed, particularly studies which control for associated lifestyle changes. The third prospective study was conducted in Japan by Naito et al. This was a single arm, open-labeled study in patients with OSA of moderate to severe degree. After 1 month of CPAP treatment, the left ventricular ejection fraction (LVEF) improved from 37.2 to 43.2%. Age and body mass index were independently associated with LVEF at the end of study with adjusted coefficients of −0.001 and 0.005, respectively. Their data indicated that an improvement in LVEF was likely to be observed in young patients with obesity. Even though the coefficients were quite small, this could be due to small sample size, necessitating need for studies with a larger sample size. Finally, Wang et al. conducted a randomized controlled trial to evaluate the effect of drug-induced sleep endoscopy (DISE)-guided CPAP titration in comparison to physician guided CPAP pressure determination on subjective daytime sleepiness in patients with moderate to severe OSA. There were no significant differences in terms of CPAP pressure, residual AHI, compliance and daytime sleepiness between the two groups. They found that epiglottis (anterior-posterior collapse) was the independent factor for 95% of CPAP pressure. They concluded that both modalities are comparable in establishing optimal CPAP pressure to treat patients.

In conclusion, OSA is frequently undiagnosed and is associated with cardiovascular risks. However, studies evaluating impact of treatment of sleep disordered breathing particularly OSA on cardiovascular risks have been limited, primarily due to poor adherence and small sample sizes. Future research needs are as follows: identification of OSA patients who are at high risk for developing cardiovascular diseases and randomized controlled trials evaluating long-term benefits of CPAP.

## Author contributions

All authors listed have made a substantial, direct, and intellectual contribution to the work and approved it for publication.

## Conflict of interest

The authors declare that the research was conducted in the absence of any commercial or financial relationships that could be construed as a potential conflict of interest.

## Publisher's note

All claims expressed in this article are solely those of the authors and do not necessarily represent those of their affiliated organizations, or those of the publisher, the editors and the reviewers. Any product that may be evaluated in this article, or claim that may be made by its manufacturer, is not guaranteed or endorsed by the publisher.
